# Salt Concentration Is Associated with Flavor Divergence and Microbial Succession in Naturally Fermented Watermelon Soybean Paste: Integrating Volatile Metabolite Profiling with Community Dynamics

**DOI:** 10.3390/foods15152767

**Published:** 2026-08-06

**Authors:** Heng Wang, Bing Yang, Zhenxia Cao, Yingjian Hou, Jing Yan, Shuanshuan Xue, Wanli Zhang, Lishui Chen

**Affiliations:** 1 Food Laboratory of Zhong Yuan, Luohe 462300, China; wangheng@zyfoodlab.cn (H.W.);; 2 Engineering Research Center for Industrial Microbial Resources Development and Application of Henan Province, Luohe 462300, China

**Keywords:** watermelon soybean paste, salt concentration, HS-GC-TOF-MS, flavor metabolites, microbial community

## Abstract

This research investigated the effects of salt concentration on the fermentation of watermelon soybean paste. Three experimental groups were constructed with gradient salt concentrations, and multi-dimensional analyses were performed on physicochemical properties, volatile aroma profiles, and microbial community succession throughout the fermentation process. Group X (lowest salt) exhibited the highest total acid (34.49 g/kg) and lowest pH (*p* < 0.05) after 60 d. Using headspace gas chromatography time-of-flight mass spectrometry (HS-GC-TOF-MS), a total of 97 volatile compounds were identified, of which 18 were defined as key flavor compounds (relative odor activity value (ROAV) > 0.1 and variable importance in projection (VIP) > 1). High-throughput sequencing demonstrated that *Proteobacteria* and *Firmicutes* represented the core bacterial communities while *Ascomycota* emerged as the dominant fungal phylum. The Shannon diversity index for bacteria ranged from 4.28 to 5.47 across groups. Aspergillus relative abundance in Group X decreased from 75.30% (30 d) to 43.17% (60 d). Group X was distinctly separated from Y and Z in PLS-DA and PCoA analyses. *Aspergillus* exhibited significant positive correlations with eugenol and 2,2,4-trimethyl-1,3-pentanediol diisobutyrate (*p* < 0.05). These findings provide an integrated understanding of the associations between volatile compound evolution and microbial succession during natural fermentation, providing a basis for further investigation and process optimization.

## 1. Introduction

Asia has a tradition of consuming fermented soybean foods since ancient times, such as Chinese soybean pastes (*Da-jiang*) [1,2], soy sauce [3], sufu [4,5] in China, miso [6,7] and natto [8,9] in Japan, *doenjang* [10,11] in Korea, and tempeh [12] in Indonesia. Fermented soybean products have gained consumer popularity due to their unique flavor, digestibility, and shelf stability. As a bean fermented soybean product in China, watermelon soybean paste serves as a time-honored condiment in traditional Chinese dishes. It is rich in beneficial proteins, vitamins and minerals, and possesses antioxidant [13], anti-hypertensive [14], anticancer and anti-microbial properties [15], which can improve anemia and regulate the human immune system [16].

Conventional soybean paste has been enhanced through production innovations. During manufacturing, sweet and succulent watermelons are incorporated, yielding a watermelon-infused soybean paste that blends the robust taste of traditional soybean paste with watermelon’s fresh, mildly sweet fragrance. The unique components in the watermelon soybean paste mainly come from the special smell produced by the fermentation of soybeans and watermelons, making the flavor rich and complex. They can not only be used as a side dish but also as an important seasoning for cooking food. With its one-of-a-kind flavor and all those benefits, watermelon soybean paste has a lot of potential for research and new ideas.

The fermentation stage is the crucial factor for the formation of watermelon soybean paste flavor. The main problems existing in the production of traditional fermented seasonings are inconsistent quality and poor hygiene conditions. During the fermentation process, salt is an important factor influencing the dynamics of microorganisms. Salt can provide osmotic pressure and inhibit the growth of foodborne spoilage bacteria. Reductions in salt concentration can disrupt normal fermentation processes and promote the proliferation of spoilage microorganisms. Compared with high-salt systems, low-salt fermentation will cause changes in the microbial composition during the fermentation process, thereby altering the flavor of the product and reducing its safety [17]. Furthermore, the reduction of sodium chloride will affect the taste and flavor of the watermelon soybean paste.

Excessive NaCl consumption can contribute to the development of hypertension and cardiovascular disorders, posing significant risks to human health. Mounting research suggests that high salt intake may also elevate the likelihood of developing gastric cancer, osteoporosis, and obesity [18]. As people gradually become aware of the dangers brought about by a high-salt diet, the development of low-salt products has become a trend [19]. The Chinese government set a goal in the National Nutrition Plan 2017–2030 to reduce the salt intake of Chinese citizens by 20% by 2030 [20]. In recent years, the use of potassium salts [21] or other substitutes (yeast extract or salty peptides) [22] has been the main approach for food manufacturers to reduce salt content in their products. However, these methods are not applicable to the production of fermented bean-based foods.

Li et al. [23] studied the effects of low salt and inoculated starters on volatile and nonvolatile substances of *doubanjiang* and found that inoculation of *T. halophilus* and *Z. rouxii* had a significant effect on volatile and nonvolatile components. The microflora structure, metabonomics and volatilomics profiles of low-salt fermented chilies were studied by a high-order positive interaction lactic acid bacteria co-fermentation interaction network [24]. Previous studies have conducted multi-omics analyses on the evolution of the microbial community during the post-fermentation process of *Doubanjiang*, as well as on the changes in flavor compounds [25]. Although salt reduction and flavor–microbiome interactions have been studied in broad bean paste and chili fermentation, there was a gap in understanding these dynamics specifically in watermelon soybean paste, which has a unique substrate composition and traditional production method.

Today, polymerase chain reaction-denaturing gradient gel electrophoresis (PCR-DGGE), pyrosequencing, 16S rDNA gene amplification, metagenomics and other biological methods have been applied to the analysis of microbial diversity. Compared with previous methods, next-generation sequencing (NGS) based on the Illumina MiSeq sequencing platform has opened up new avenues for exploring the structure and diversity of microorganisms and has been widely applied in the analysis of microbial diversity in many fermented foods. Nevertheless, data on microbial community variations and flavor-active compound profiles in traditionally fermented watermelon soybean paste remain scarce. High-throughput sequencing technology combined with bioinformatics methods has been used to analyze microbial community composition and function during watermelon soybean paste fermentation, aimed to reveal patterns and functions of microbial succession across major fermentation stages, enrich the research on the flavor change of soybean paste and the composition of fermented microbial community, and provide a theoretical basis for improving the quality of soybean paste.

In this study, therefore, the combined method was adopted to investigate the evolution of volatile organic compounds and microorganisms in soybean paste with different salt contents, and the results could provide valuable information for a deeper understanding of the fermentation process, improving its flavor, and enhancing product quality.

## 2. Materials and Methods

### 2.1. Sample Preparation

Watermelon soybean paste production consisted of two stages: koji preparation and fermentation. The soybeans (purchased from a local market in Luohe, Henan, China) were soaked in distilled water (m:v = 1:4), then cooked for 15 min. The koji was prepared by inoculating cooked soybeans with a commercial koji starter (*Aspergillus oryzae*) at an inoculation rate of 0.02% (*w*/*w*). The inoculated soybeans were spread and incubated at 32 ± 2 °C for 48 h with periodic turning to maintain aerobic conditions and uniform temperature. After 48 h, the koji exhibited visible mycelial growth, indicating successful fermentation.

The fresh watermelon juice was prepared by homogenizing ripe watermelon (purchased from a local market in Luohe, Henan, China) and filtering through gauze to remove seeds. Spice water was prepared by boiling a mixture of water, star anise, Sichuan pepper, cinnamon, bay leaf, and ginger (ratio of spices to water = 1:20, *w*/*v*) for 30 min. The koji (2 kg per batch) was mixed separately with salt (m:m = 1:0.2, 1:0.3, 1:0.4), watermelon juice (m:m = 1:3), spice water (m:m = 1:0.2), and the respective amount of salt in a glass fermentation vessel. The experimental groups with different salt additions were labeled as X (m:m = 1:0.2), Y (m:m = 1:0.3), and Z (m:m = 1:0.4), respectively. The fermentation period extended for 60 d. Fermentation temperatures were controlled at 42 °C for 15 d, followed by 32 °C for 45 d, and humidity was maintained at 70–80% by placing water trays in the incubator. Samples were stirred every three days throughout the fermentation period. Sampling times were selected to represent distinct stages of fermentation: 30 d corresponded to early maturation after the temperature transition from 42 °C to 32 °C, 45 d represented the intermediate fermentation stage, and 60 d represented the final product. Earlier time points were not included because the characteristic volatile profile was not yet fully developed during the initial temperature-controlled phase.

Samples of the fermentation batches per treatment per time point (30, 45 and 60 d) were collected for analysis. Specifically, for each treatment (X, Y, Z), three independent fermentation vessels were prepared. The experimental design used independent fermentation vessels for each time point. All the above were sampled at multiple points and homogenized, and then stored in the refrigerator at −80 °C for sample delivery and standby.

### 2.2. Measurement of Physicochemical Parameters

The proximate chemical composition of moisture, total lipid, crude protein, total ash, amino acid nitrogen and NaCl content of all samples were determined according to the Chinese National Standards: GB5009.3–2016 [26], GB5009.6–2016 [27], GB5009.5–2016 [28], GB5009.4–2016 [29], GB 5009.235–2016 [30] and GB 5009.44-2016 [31], respectively.

According to the national standard GB12456-2021 [32], the total acid concentration was measured by sodium hydroxide titration. For the pH measurements of watermelon soybean paste, 5 g of each sample was diluted in 45 mL of distilled water, followed by homogenization, soaking for 30 min and centrifuging at 4000 r/min for 5 min. The pH of the sample was measured using a pH meter (METTLER TOLEDO, Shanghai, China).

### 2.3. Gas Chromatography Time-of-Flight Mass Spectrometry (GC-TOF-MS) Analysis

The volatile compounds in the samples collected after 30, 45 and 60 d of fermentation were determined by GC-TOF-MS. A 2 g sample was accurately weighed into a headspace vial, 2 g NaCl and 8 mL distilled water were added, and the vial was immediately sealed. The headspace vial was equilibrated at 60 °C for 30 min, and then the volatile compounds in the headspace vial were extracted using an SPME fiber: a 50/30 μm DVB/CAR/PDMS fiber (Supelco, Bellefonte, PA, USA). The head was subsequently transferred to the injection port at 250 °C for desorption for 5 min.

GC-TOF-MS analyses were performed on an Agilent 8890 GC system equipped with an Agilent 7250 Precision Mass QTOF mass spectrometer (Agilent Technologies, Palo Alto, CA, USA). GC conditions: the column was HP-5MS (15 m × 0.25 mm × 0.25 μm), ramp-up procedure: initial column temperature was 40 °C, held for 2 min, ramped up to 180 °C at 5 °C/min, held for 2 min, then ramped up to 240 °C at 12 °C/min, and held for 5 min; MS conditions: scan range *m*/*z* 35–500; scan rate 10 scans/s, transfer line temperature 250 °C, ion source temperature 200 °C, ion source energy 70 eV.

Volatile compounds were identified by matching mass spectra with the NIST 2020 mass spectral library (match threshold > 80%). No internal standard was used because volatile compounds were evaluated by relative peak-area normalization. Retention-index confirmation was not performed; therefore, compound assignments should be regarded as putative identifications based on mass-spectral similarity. The relative abundance of volatile compounds was estimated using peak-area normalization.

### 2.4. Calculation of Relative Odor Activity Values

The relative odor activity values were calculated by the formula:

$$\mathrm{RQAV}\;=\;\frac{C_{i}}{T_{i}}\times \frac{T_{\max}}{C_{\max}}\times 100$$

where Ci is the relative abundance of a volatile compound, %; Ti is the sensory threshold of a volatile compound, μg/kg; the relative abundance of volatile compounds with the largest contribution of Cmax to flavor, %; and Tmax is the sensory threshold of the volatile compounds that contribute the most to flavor, μg/kg.

The ROAV was used to evaluate the contribution of each volatile component to the flavor samples, in which ROAV  >  1 indicates that the component contributes the most to the sample’s flavor, and is the key flavor component; 0.1  <  ROAV  <  1 indicates that the component can change the sample’s flavor; and ROAV  <  0.1 indicates that the component does not have a significant effect on the sample’s flavor [33].

### 2.5. Total DNA Extraction, PCR Amplification and Illumina MiSeq Sequencing

Total genomic DNA was extracted from individual samples from independent biological replicates using a MagBeads FastDNA Kit for Soil (116564384) (MP Biomedicals, Santa Ana, CA, USA). The integrity of genomic DNA was judged by 0.8% (*w*/*v*) agarose gel electrophoresis, and then it was accurately quantified by ultraviolet quantitative equipment (NC 2000 UV Quantitative Equipment, Thermo Scientific, Carlsbad, CA, USA).

PCR primers of V3-V4 hypervariable region of 16S rRNA gene of bacteria were 338F (5′- barcode+ACTCCTACGGGAGGCAGCA-3′) and 806R (5′-GGACTACHVGGGTWTCTAAT-3′). For the fungi ITS rRNA gene, primers ITS5 (GGAAGTAAAAGTCGTAACAAGG) and ITS2 (GCTGCGTTCTTCATCGATGC) were amplified.

The PCR reaction mixture was: 0.25 μL Q5 high-fidelity DNA polymerase (M0491L Q5^®^ High-Fidelity DNA Polymerase, NEB), 5 μL 5× Reaction Buffer, 5 μL 5× High GC Buffer, 2 μL dNTP (10 mM), 2 μL template DNA, 1 μL each forward and reverse primers (10 μM), and 8.75 μL deionized water. PCR condition was conducted as follows: initial denaturation at 98 °C for 5 min; 25 cycles of denaturation at 98 °C for 30 s, annealing at 52 °C for 30 s, elongation at 72 °C for 45 s; a final extension at 72 °C for 5 min; finally maintained at 12 °C. The quality of the PCR products was examined by electrophoresis using agarose gel (2%).

Amplification and libraries constructed were provided by Personal Biotechnology Co., Ltd. (Shanghai, China). Finally, PCR-purified products were mixed and sequenced on an Illumina MiSeq platform using paired-end sequencing (Illumina, San Diego, CA, USA).

Sequencing depth (30,000–55,000 reads), quality filtering parameters (DADA2: truncLen 220/200, maxEE 2), and rarefaction depth (30,000).

### 2.6. Sequence Analysis

The raw data was filtered, the barcodes removed, and the sequence denoising and amplicon sequence variant (ASV) generation using DADA2 were carried out by using QIIME2 software (Quantitative Insights Into Microbial Ecology, v2024.5, http://qiime.org/). QIIME2 was used to classify and annotate the taxonomic information for each ASV representative sequence based on Greengenes 2 database for bacteria (Release 13.8, http://greengenes.secondgenome.com/) and the UNITE database for fungi (Release 8.0, https://unite.ut.ee/). Alpha diversity and beta diversity were calculated for 9 samples. Greengenes 2 was used because it was the database implemented by the sequencing service provider during the original analysis. The use of this database represents a methodological limitation; future analyses should consider current curated databases such as SILVA to improve taxonomic resolution and comparability. Sequencing data have been deposited in the NCBI Sequence Read Archive (SRA) under BioProject accession number PRJNA1498591.

### 2.7. Statistical Analysis

For each biological sample, physicochemical parameters and GC-TOF-MS volatile profiles were measured in analytical triplicate, and results are presented as mean ± standard deviation, with the exception of microbial community relative abundance data (n = 6). At each fermentation time point, using SPSS statistical software (version 26.0; IBM, Armonk, NY, USA), the differences among salt treatments were analyzed by separately using one-way ANOVA followed by Duncan’s multiple-range test (*p* < 0.05). Origin 2021 (MicroCal, Northampton, MA, USA) and SIMCA 14.1 (MKS Umetrics, Umea, Sweden) were used for data processing and graphing. Spearman’s correlation analysis was used to evaluate the correlation between bacterial and fungal communities and key volatile flavor compounds.

## 3. Results and Discussion

### 3.1. Physicochemical Parameters

#### 3.1.1. The Proximate Chemical Compositions

The proximate chemical compositions of watermelon soybean paste (60 d) from different salt contents are shown in Table 1. There were significant differences in the NaCl content at the end of fermentation among the three groups (*p* < 0.05). Moisture content differed significantly among salt treatments (*p* < 0.05), with Group X showing the lowest moisture content. Fat and protein contents also differed significantly among treatments (*p* < 0.05); Group X exhibited the highest fat (10.67 g/100 g) and protein (22.45 g/100 g) contents at 60 d. The results of ash content were contrary to those of fat and protein, and Group Z was the highest, which was 20.50 g/100 g.

#### 3.1.2. Amino Acid Nitrogen

Amino acid nitrogen content serves as an indicator of protein hydrolysis degree and can reflect the fermentation maturity of watermelon soybean paste. As shown in Table 1, significant differences in amino acid nitrogen levels were observed among watermelon soybean paste samples with varying salt concentrations. Inoculation with *Aspergillus oryzae* during fermentation was found to significantly boost amino acid nitrogen production. The growth of *Aspergillus oryzae* can provide the fermentation with abundant proteases, lipases and amylases. In this study, when the fermentation process reached 60 d, sample Y had the highest amino acid nitrogen content, which was 1.75 g/100 g. There were significant differences in amino acid nitrogen content among the three groups of samples (*p* < 0.05). The differences in amino acid nitrogen content among the three groups may be associated with differential protease activity under varying salt concentrations, as *Aspergillus oryzae*-derived proteases are known to be salt-sensitive. However, as protease activity was not directly measured in this study, this interpretation remains to be verified experimentally.

#### 3.1.3. Total Acid and pH

**Figure 1:**
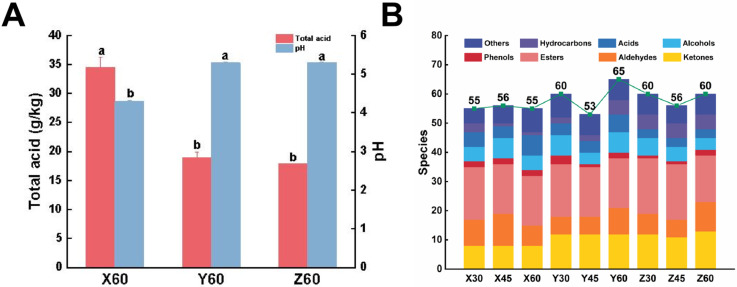
Total acid and pH of watermelon soybean paste from different salt contents (**A**). The species of volatile aroma compounds of watermelon soybean paste from different salt contents (**B**). Note: Values marked with lower case letters in the same line were significantly different (*p* < 0.05).

Total acid is an important indicator for evaluating the quality of watermelon soybean paste. It participates in the Maillard reaction and esterification reaction during the fermentation process and has a significant impact on the formation of the color and flavor. The total acid and pH of watermelon soybean paste from different salt contents are depicted in Figure 1A.

After 60 d of fermentation, the total acid content of Group X was the highest, which was 34.49 g/kg, which may be caused by the decrease of water content in the later stage of fermentation, which affected the metabolic activity of acid-producing microorganisms. The total acid in watermelon soybean paste mainly originates from the acidic metabolites produced by microbial activity, as well as from microbial cell autolysis and free amino acid accumulation resulting from fermentation environment fluctuations. The pH value of Group X was significantly lower than that of the other two groups (*p* < 0.05), while there was no significant difference in pH between Y and Z (*p* > 0.05). The results of total acid content and pH value confirm each other.

### 3.2. GC-TOF-MS Analysis

As shown in Table 2, a total of 97 volatile compounds were detected by GC-TOF-MS, including 14 ketones, 17 aldehydes, 23 esters, 4 phenols, 9 alcohols, 10 acids, 6 hydrocarbons, and 14 other compounds. These components exist in a balanced state, complement each other, and collectively form the unique aroma flavor of soybean paste. The species of volatile aroma compounds of watermelon soybean paste from different salt contents range from 53 to 65 (Figure 1B). The species of volatile aroma compounds in Y change the most drastically. Collectively, the experimental group with elevated salt concentrations exhibited a more diverse profile of volatile aroma compounds in the final fermentation product.

Table 2 shows the detailed information of the major flavor compounds identified in samples. Throughout the fermentation process, the levels of aldehydes and alcohols in Group X decreased significantly, while the ketone content remained relatively stable between 30 d and 45 d of fermentation. In Group Y, the contents of alcohols and acids increased significantly (*p* < 0.05), while the contents of esters decreased. In Group Z, the contents of ketones and aldehydes increased, while the contents of alcohols decreased.

On the 30 d, the ketones and aldehydes in Group X were significantly higher (*p* < 0.05) than those in Groups Y and Z. During the middle stage (45 d), ketone (20.39%) and acid (5.95%) contents of Y were the highest, and phenols (0.59%) and hydrocarbons (0.80%) were the lowest. At 60 days, the contents of aldehydes (24.65%), esters (22.70%) and hydrocarbons (4%) in Group Z were the highest, while the contents of phenols (1.75%), alcohols (13.03%) and acids (4.03%) were the lowest.

Acetophenone was considered a contributor to the fishy and musty smell of beans, and only existed in the Y and Z groups. Benzophenone, characterized by rose and camphor-like aromatic notes, was detected in all samples, but the relative abundance was lowest at the final fermentation stage of Group Y, reaching only 0.03%. Aldehyde compounds were considered to be the most important compounds to form the flavor of fermented bean products, because they could emit the flavor of grass, nuts, candied fruits and cheese. In the fermentation process, the relative abundance of aldehydes in Group X was completely different from that in Groups Y and Z, with a noticeable decrease over time. After 60 d of fermentation, the total content of aldehyde substances was the lowest in Group X (13.53%) and the highest in Group Z (24.65%). Moreover, there were significant differences among the samples of each group (*p* < 0.05). Higher salt treatments were associated with greater relative abundance of aldehydes at 60 d.

At 60 days, the contents of palmitic acid ethyl ester and linoleic acid ethyl ester increase with the increase of salt content, while ethyl oleate only appears in the X45 and Y60. Palmitic acid ethyl ester, linoleic acid ethyl ester, and ethyl oleate are all produced during the fermentation of beans [34]. They have a typical fruity aroma, which makes the aroma of watermelon soybean paste more harmonious, rich, balanced and coordinated. At the end of the fermentation process, the content of esters in Group Z was the highest (22.70%), but there was no significant difference between Groups X and Y (*p* > 0.05). In terms of the composition of the odor, ester substances make the overall odor gentler and enhance the complexity of the final flavor profile. Overall, salt concentration was associated with marked differences in the volatile profile of watermelon soybean paste.

Alcohols play a role in forming a mellow flavor and providing precursors for the synthesis of esters. Linalool gives fresh, woody and citrus aromas [35]. There are relatively few acids in the sample, and it has been reported that acids will have a negative impact on the flavor of fermented bean products (such as cheonggukjang and Japanese natto), making it taste poor [36]. At 60 d, Group X showed the highest relative abundance of acids (19.52%). This profile may be associated with less desirable sensory attributes reported for highly acidic fermented soybean products; however, sensory acceptability was not directly evaluated in the present study. It is consistent with the results of pH and total acid. 2-Acetylpyrrole has a nutty aroma and can be found in tea, coffee, and roasted beef. Pyrazine is mainly formed by the Maillard reaction involving protein/amino acid and reducing sugar, which often gives aroma to soil, nuts and baking.

Some compounds detected in the samples, including certain phthalates, may originate from laboratory or packaging contamination rather than from the fermentation process itself. Because procedural blanks and contamination controls were not included in the analytical procedure, the origin of these compounds cannot be definitively established from the current data. Accordingly, these compounds should not be interpreted as genuine flavor constituents without confirmatory evidence, and they were not included in the key flavor compound analysis based on ROAV and VIP criteria.

It should be noted that peak area normalization provides relative abundance data, not absolute quantification. While this approach enables comparison of compound profiles across samples, absolute concentrations would require calibration curves with authentic standards. Future studies should employ quantitative methods such as stable isotope dilution or standard addition for accurate quantification of key flavor compounds.

### 3.3. PLS-DA Analysis of Volatile Compounds

**Figure 2:**
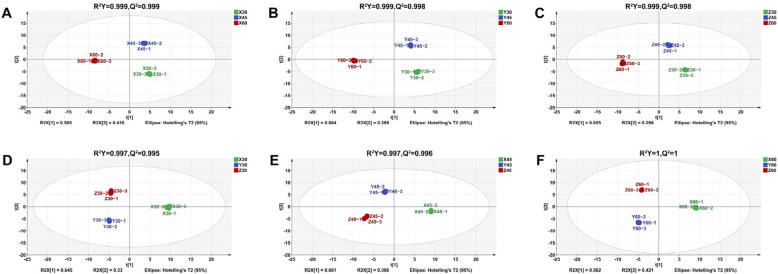
PLS-DA analysis of volatile aroma compounds of watermelon soybean paste from different salt contents. ((**A**): PLS-DA of Group X of watermelon soybean paste in three fermentation stages (30 d, 45 d, 60 d), (**B**): PLS-DA of Group Y of watermelon soybean paste in three fermentation stages (30 d, 45 d, 60 d), (**C**): PLS-DA of Group Z of watermelon soybean paste in three fermentation stages (30 d, 45 d, 60 d), (**D**): PLS-DA of 30 d of watermelon soybean paste from different salt contents, (**E**): PLS-DA of 45 d of watermelon soybean paste from different salt contents, (**F**): PLS-DA of 60 d of watermelon soybean paste from different salt contents).

Partial Least Squares Discriminant Analysis (PLS-DA) is a supervised discriminant statistical technique that demonstrates distinct advantages in handling complex high-dimensional datasets, making it particularly effective for analyzing volatile organic compounds. Figure 2 and Figure S1 present the PLS-DA results, illustrating differences in watermelon soybean paste with varying salt concentrations across different fermentation periods. Figure 2A–C show the PLS-DA results of the watermelon soybean paste with different salt contents (X(A), Y(B), Z(C)) at three fermentation stages (30 d, 45 d, 60 d). Among all the samples, 30 d was located primarily in the fourth quadrant, 45 d was mainly in the first quadrant, and 60 d was mainly on the negative side of the *x*-axis (Figure 2A–C). Watermelon soybean paste samples demonstrated relatively uniform distribution across different salt concentration gradients, suggesting minimal inter-group variability. However, the larger distances among different fermentation times showed significant differences between groups. The 30 and 45 d groups were spatially close to each other, while the 60 d group was farther apart. This indicated that the volatile compound profile of the 60 d group was more distinct than those of the 30 and 45 d groups. Figure 2D–F showed the PLS-DA results combining different salt contents (X, Y, Z) within one fermentation stage (30 d (D),45 d (E),60 d (F)). The samples in each part were closely clustered, indicating that there is little intra-group variation among samples. Specifically, Group X was primarily positioned along the positive *x*-axis, Group Y was mostly found in the third quadrant, and Group Z was predominantly located in the second quadrant. Further analysis revealed that Group Z and Group Y exhibited closer clustering, whereas Group X was distinctly separated from both. These results indicated that the difference between Group X and the other two groups was greater.

### 3.4. Screening of the Key Volatile Compounds

**Figure 3:**
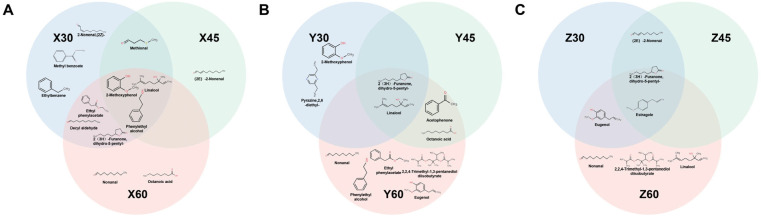
The key volatile compounds of watermelon soybean paste from different salt contents ((**A**): Group X, (**B**): Group Y, (**C**): Group Z).

A total of 97 volatiles (Table 2) were identified, undoubtedly, only some played key roles in forming product flavor. We calculated ROAV values for volatile substances and distinguished key volatile compounds based on the ROAV magnitude. Furthermore, based on the PLS-DA analysis, the volatile compounds with VIP > 1 were also identified as the key flavor compounds of the watermelon soybean paste. Based on integrated analyses, volatile flavor compounds with ROAV > 0.1 and VIP > 1 were identified as significant contributors to the flavor of watermelon soybean paste. The key volatile compounds included in each group of samples are shown in Figure 3.

There were 18 key flavor compounds in the watermelon soybean paste with different salt contents, namely, 2-nonenal,(2Z)-, decyl aldehyde, methyl benzoate, ethyl phenylacetate, 2(3H)-furanone,dihydro-5-pentyl-, 2-methoxyphenol, linalool, phenylethyl alcohol, ethylbenzene, methional, (2E)-2-nonenal, nonanal, octanoic acid, pyrazine, 2,6-diethyl-, acetophenone, 2,2,4-trimethyl-1,3-pentanediol diisobutyrate, eugenol, estragole.

In Group X, 2-methoxyphenol, linalool, and phenylethyl alcohol were present throughout the process. Meanwhile, 2-nonenal, methyl benzoate, and ethylbenzene were only detected for 30 d. (2E)-2-nonenal was only present for 45 d, and nonanal and octanoic acid were only present for 60 d. In Group Y, 2(3H)-furanone, dihydro-5-pentyl- and linalool were detected at all three fermentation time points. In contrast, 2-methoxyphenol and pyrazine, 2,6-diethyl- were only detected on 30 d, while acetophenone and octanoic acid were detected on both 45 and 60 d.

In Group Z, 2(3H)-furanone, dihydro-5-pentyl- and estragole were detected throughout the entire process. (2E)-2-nonenal was detected on both 30 and 45 d, and eugenol was detected by 60 d. Nonanal, 2,2,4-trimethyl-1,3-pentanediol diisobutyrate, and linalool were only detected on 60 d.

These indicated a significant variation in the contribution of key flavor compounds to different experimental samples, resulting in pronounced flavor disparities among them.

### 3.5. Microbial Community Composition Analysis

Microorganism number, nature, and distribution determine fermentation speed and fermented product quality. Therefore, a thorough understanding of the relationship between microbial community and aroma compounds is a prerequisite for producing high-quality watermelon soybean paste through fermentation. The sequence coverage rate is greater than 0.999, indicating that the authenticity and depth of sequencing are sufficient. Alpha diversity indexes can reflect microbial diversity (Shannon, Simpson) and abundance (Chao1) [37]. The greater the value of each index in Table 3, the higher the diversity. The index of bacteria and fungi showed that the abundance of bacteria in watermelon soybean paste was higher than that of fungi, which indicated that the diversity of bacteria in watermelon soybean paste was more abundant. The results displayed that the bacterial diversity of Group X was lower than that of Y and Z, while the fungal diversity was higher. The low-salt treatment was associated with lower bacterial diversity and higher fungal diversity under the conditions evaluated in this study.

**Figure 4:**
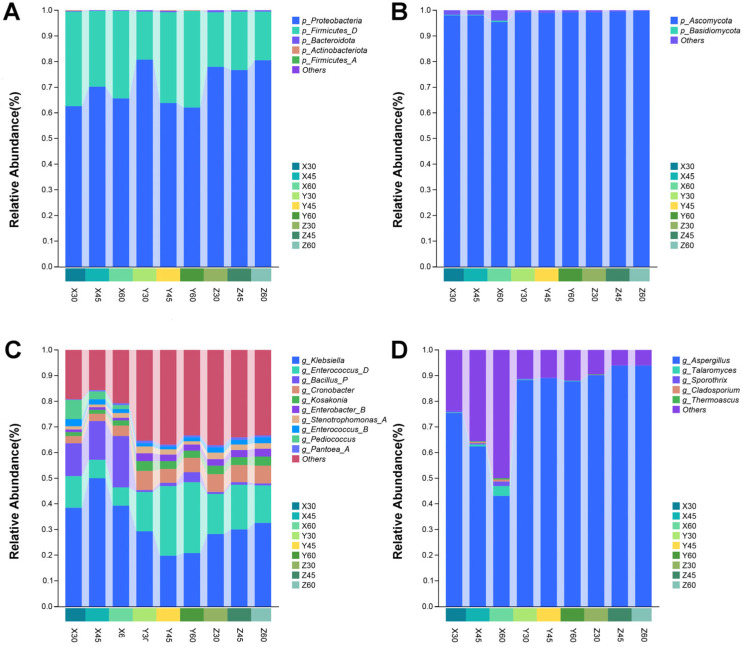
Taxonomic classification of the bacterial 16S rRNA and fungal ITS2 sequences at the phylum and genus level showing bacterial and fungal community changes of watermelon soybean paste from different salt contents ((**A**): bacterial phyla, (**B**): fungal phyla, (**C**): bacterial genera and (**D**): fungal genera).

Figure 4 illustrates the dynamic changes in bacterial and fungal communities at both the phylum and genus levels throughout the fermentation of watermelon soybean paste. Among bacteria, *Proteobacteria* and *Firmicutes* were identified as the dominant phyla during the fermentation process. In addition, some phyla with low abundance, such as *Bacteroidota* and *Actinobacteriota*, were detected, and their relative abundance was less than 1% in the whole fermentation process. *Ascomycota*, *Proteobacteria* and *Firmicutes* were important components of microbial flora in Guangdong Cantonese soy sauce, *Donajiang* and Dajiang in South Korea, which showed that they play an important role in producing high-quality traditional fermented soybean food. The *Klebsiella* (19.75–50.06%), *Enterococcus_D* (7.01–27.62%), *Bacillus_P* (0.71–20.02%), *Cronobacter* (2.73–7.46%), *Kosakonia* (1.51–3.93%), *Enterobacter_B* (0.96–3.08%), *Stenotrophomonas_A* (1.03–2.77%), *Enterococcus_B* (1.11–2.70%), and *Pediococcus* (0–7.33%) were dominant genera during the fermentation (Figure 4C). The relatively high abundance of *Klebsiella* in Group X (low-salt treatment) warrants attention because members of this genus may include undesirable microorganisms in fermented foods. However, amplicon sequencing alone does not establish viability or food-safety risk, and targeted microbiological analyses would be required. The lower relative abundance of *Klebsiella* in Groups Y and Z is consistent with the inhibitory effect of high osmotic pressure reported in previous studies [24].

Figure 4B shows that the fungi detected during watermelon soybean paste fermentation mainly belonged to the phyla *Ascomycota* and *Basidiomycota*, with a combined relative abundance of over 97%. In the whole fermentation cycle, *Ascomycota* was absolutely dominant, and the abundance of Group X decreased slightly after 60 d of fermentation. It was found that *Aspergillus* was the dominant genus of fungi. In addition, the *Aspergillus* genus is also regarded as the key functional microorganism in the fermentation process of Chinese bean products such as doubanjiang [38] and sufu [39]. The relative abundance of *Aspergillus* in Group X decreased gradually with the increase of fermentation time (X30:75.30%, X45:62.27%, X60:43.17%). It can secrete various enzymes that facilitate raw material decomposition and is involved in esterification and glycolysis during fermentation [40].

### 3.6. Beta Diversity and Dominant Species for Microbial Community

**Figure 5:**
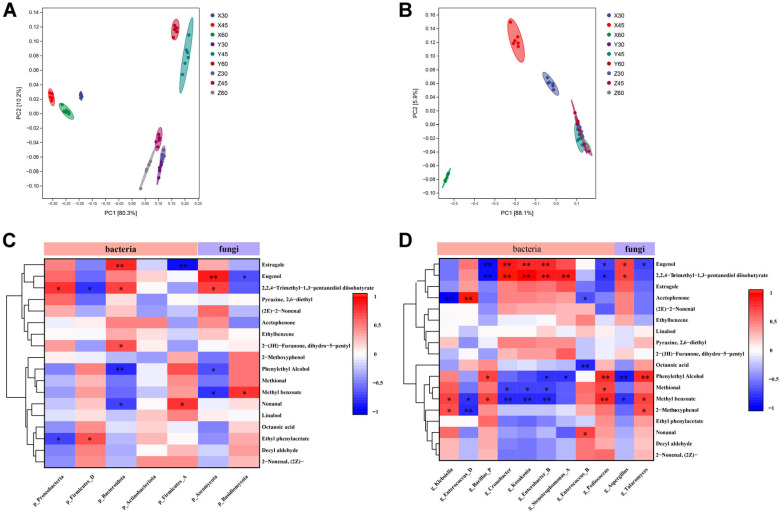
PCoA score plot of watermelon soybean paste from different salt contents ((**A**): Bacteria, (**B**): Fungi). Correlation between microbial abundance and major volatile flavor compounds at the level of phylum (**C**) and genus (**D**). * 0.01 < *p* ≤ 0.05, ** 0.001 < *p* ≤ 0.01.

In this study, the similarity and difference of microbial populations among different samples during the fermentation of watermelon soybean paste were evaluated by the distance between each sample point in the principal coordinate analysis diagram (PCoA). PCo1 and PCo2 contributed 80.3% and 10.2% of the difference, respectively, a total of 90.5% (Figure 5A). Y30 was close to Group Z, with little species difference, but far from Y45 and Y60, with great species difference, indicating that in the early stage of fermentation, Group Y was close to Group Z, and the microbial community succession of Group Y and Group Z had undergone different changes in the fermentation stage from 30 to 60 days. Generally speaking, Groups X, Y and Z with different salt contents are well distinguished. The first two principal components account for 88.1% and 5.9% of the variance contribution rate in the fungal community. Principal coordinate analysis of fungi showed that X30, X45, X60 in Group X were far apart, with great species differences, while Group Y and Group Z were competing with each other, with little species differences.

### 3.7. Correlation Between Key Volatile Compounds and Microbial Abundance Analysis

Flavor compound formation is tightly coupled with microbial activity, as evidenced by the complex and interdependent relationships between microbial communities and flavor development. In Figure 5C,D, the relationship between key volatile compounds and microbes was analyzed. In bacteria, *Proteobacteria* and *Firmicutes-D* showed significant correlations with 2,2,4-trimethyl-1,3-pentanediol diisobutyrate and ethyl phenylacetate (*p* < 0.05). The *Bacteroidota* also exhibited significant associations with five volatile organic compounds: estragole, 2,2,4-trimethyl-1,3-pentanediol diisobutyrate, 2(3H)-furanone,dihydro-5-pentyl-, phenylethyl alcohol, and nonanal. In contrast, the *Actinobacteriota* did not show any significant correlations with the 18 volatile substances examined (*p* > 0.05). *Firmicute-A* was significantly correlated with estragole and nonanal. Among fungi, *Ascomycota* was significantly correlated with eugenol, 2,2,4-trimethyl-1,3-pentanediol diisobutyrate, phenylethyl alcohol, and methyl benzoate (*p* < 0.05). *Basidiomycota* was only significantly correlated with eugenol and methyl benzoate (Figure 5C).

*Klebsiella* was significantly positively correlated with methyl benzoate and 2-methoxyphenol, while showing a significant negative correlation with acetophenone. In contrast, *Enterococcus_D* displayed an inverse correlation with these three volatile compounds. *Cronobacter*, *Kosakonia*, *Enterobacter_B* were significantly positively correlated with eugenol, 2,2,4-trimethyl-1,3-pentanediol diisobutyrate, and significantly negatively correlated with methyl benzoate and methional (*p* < 0.05). *Bacillus_P* and *Pediococcus* showed a significant negative correlation with eugenol, 2,2,4-trimethyl-1,3-pentanediol diisobutyrate, and a positive correlation with phenylethyl alcohol and methyl benzoate (*p* < 0.05). Among fungi, *Aspergillus* showed a positive correlation with eugenol, and a negative correlation with phenylethyl alcohol and methyl benzoate (*p* < 0.05). In contrast, *Talaromyces* exhibited the opposite pattern (Figure 5D). Microbial community succession was significantly associated with changes in the composition and relative abundance of volatile compounds during fermentation. Salt concentration influences flavor through both direct (e.g., taste perception, enzyme activity) and indirect (e.g., microbial community selection) pathways. While our data demonstrate associations between salt concentration, microbial composition, and volatile profiles, disentangling direct from indirect effects would require controlled experiments with defined microbial communities.

## 4. Conclusions

This study showed that salt concentration was significantly associated with differences in physicochemical properties, volatile profiles, and microbial community succession in watermelon soybean paste. Following 60 days of fermentation, Group X (lowest salt concentration) exhibited the lowest moisture content, the highest total acid concentration, and the lowest pH value. GC-TOF-MS was used to identify volatile compounds and estimate their relative abundance across the three salt treatments. Among them, a total of 97 volatile flavor compounds were detected, of which 18 were key flavor compounds. Compared with the other two groups (Y, Z), Group X displayed three predominant volatile compounds—2-methoxyphenol, linalool, and phenylethyl alcohol—during fermentation, which were responsible for its characteristic aroma. Microbiological results revealed that the dominant bacterial genera were mainly composed of *Klebsiella*, *Enterococcus_D*, *Bacillus_P*, *Cronobacter*, *Kosakonia*, *Enterobacter_B*, *Stenotrophomonas_A*, *Enterococcus_B*, and *Pediococcus*. *Ascomycota* were the dominant fungal taxa. *Proteobacteria* were positively correlated with 2,2,4-trimethyl-1,3-pentanediol diisobutyrate and negatively correlated with ethyl phenylacetate (*p* < 0.05), whereas *Firmicutes_D* showed the opposite pattern. *Ascomycota* were positively correlated with eugenol and 2,2,4-trimethyl-1,3-pentanediol diisobutyrate (*p* < 0.05).

This study has several limitations. First, the study was primarily correlative and did not establish causal relationships between specific microorganisms and volatile compounds. Second, protease and lipase activities were not directly measured. Despite these limitations, the findings have practical implications for the watermelon soybean paste industry. The observed differences among salt treatments provide useful information for designing salt-reduction strategies; however, determination of an optimal salt concentration will require targeted sensory, microbiological safety, and process-validation studies. The identified key flavor compounds and their microbial associations can serve as targets for process optimization and starter culture development.

## Figures and Tables

**Table 1:** Proximate chemical composition of watermelon soybean paste (60 d) from different salt concentration groups (g/100 g).

	Moisture	Fat	Protein	Ash	Amino Acid Nitrogen	NaCl
**X60**	28.84 ± 1.39 ^b^	10.67 ± 0.44 ^a^	22.45 ± 0.28 ^a^	13.76 ± 0.06 ^c^	1.69 ± 0.03 ^b^	10.34 ± 0.11 ^c^
**Y60**	32.94 ± 1.34 ^a^	9.21 ± 0.42 ^b^	17.61 ± 0.14 ^b^	18.77 ± 0.14 ^b^	1.75 ± 0.01 ^a^	15.61 ± 0.18 ^b^
**Z60**	33.29 ± 1.94 ^a^	8.09 ± 0.12 ^c^	16.25 ± 0.52 ^c^	20.50 ± 0.19 ^a^	1.52 ± 0.02 ^c^	18.65 ± 0.09 ^a^

Note: Data are mean ± standard deviation (n = 3); data in the same column with different superscripts are significantly different (*p* < 0.05).

**Table 2:** Determination of volatile substances of watermelon soybean paste from different salt contents.

Count	CAS	Compounds	Formula	RT (min)	Relative Abundance (%)								
					X30	X45	X60	Y30	Y45	Y60	Z30	Z45	Z60
1	19322-27-1	3(2H)-Furanone, 4-hydroxy-5-methyl-	C_5_H_6_O_3_	12.53	-	-	1.70 ± 0.18 ^a^	-	-	1.25 ± 0.06 ^b^	-	-	1.26 ± 0.02 ^b^
2	98-86-2	Acetophenone	C_8_H_8_O	13.13	-	-	-	0.71 ± 0.01 ^C,b^	10.78 ± 0.65 ^A,a^	3.85 ± 0.19 ^B,a^	0.92 ± 0.05 ^A,a^	0.55 ± 0.01 ^B,b^	0.40 ± 0.03 ^C,b^
3	821-55-6	2-Nonanone	C_9_H_18_O	13.92	3.71 ± 0.0 ^B,a^	4.38 ± 0.22 ^A,a^	3.13 ± 0.22 ^C,a^	0.76 ± 0.03 ^B,c^	1.94 ± 0.19 ^A,c^	-	1.33 ± 0.12 ^B,b^	2.36 ± 0.19 ^A,b^	1.52 ± 0.13 ^B,b^
4	118-71-8	Maltol	C_6_H_6_O_3_	14.46	5.22 ± 0.20 ^A,a^	4.63 ± 0.17 ^B,b^	0.99 ± 0.06 ^C,c^	3.50 ± 0.11 ^A,b^	2.84 ± 0.05 ^B,c^	2.44 ± 0.19 ^C,b^	1.97 ± 0.12 ^C,c^	5.48 ± 0.27 ^A,a^	4.10 ± 0.21 ^B,a^
5	1125-21-9	2,6,6-Trimethyl-2-cyclohexene-1,4-dione	C_9_H_12_O_2_	15.47	0.95 ± 0.0 ^B,a^	1.23 ± 0.09 ^A,a^	0.20 ± 0.01 ^C,c^	0.30 ± 0.02 ^B,b^	0.28 ± 0.02 ^B,b^	0.60 ± 0.04 ^A,b^	0.12 ± 0.01 ^C,c^	0.28 ± 0.01 ^B,b^	1.46 ± 0.04 ^A,a^
6	122-00-9	4′-Methylacetophenone	C_9_H_10_O	16.67	-	-	-	0.13 ± 0.01 ^A,a^	0.15 ± 0.02 ^A^	-	0.09 ± 0 ^b^	-	-
7	110-13-4	2,5-Hexanedione	C_6_H_10_O_2_	18.34	0.11 ± 0 ^B,b^	0.31 ± 0.02 ^A,a^		0.17 ± 0.01 ^C,a^	0.19 ± 0.01 ^B,b^	0.24 ± 0.01 ^A,b^	0.11 ± 0.01 ^B,b^	0.12 ± 0 ^B,c^	0.45 ± 0.03 ^A,a^
8	5355-63-5	3-Hydroxy-4-phenyl-2-butanone	C_10_H_12_O_2_	21.13	-	-	-	0.55 ± 0.01 ^A,a^	0.27 ± 0.02 ^B,a^	0.06 ± 0 ^C,b^	0.11 ± 0.01 ^C,b^	0.17 ± 0 ^B,b^	0.25 ± 0.02 ^A,a^
9	122-84-9	4-Methoxyphenylacetone	C_10_H_12_O_2_	22.15	0.69 ± 0.07 ^B,c^	0.40 ± 0.04 ^C,b^	0.85 ± 0.05 ^A,b^	1.52 ± 0.12 ^A,a^	1.21 ± 0.13 ^B,a^	1.71 ± 0.02 ^A,a^	1.11 ± 0.05 ^A,b^	1.11 ± 0.09 ^A,a^	0.67 ± 0.05 ^B,c^
10	91-64-5	Coumarin	C_9_H_6_O_2_	23.53	0.03 ± 0 ^C,c^	0.11 ± 0 ^B,c^	0.33 ± 0.03 ^A,b^	0.41 ± 0.01 ^B,a^	0.19 ± 0 ^C,b^	0.50 ± 0.03 ^A,a^	0.25 ± 0.02 ^B,b^	0.31 ± 0.01 ^A,a^	0.21 ± 0.01 ^C,c^
11	3796-70-1	Geranylacetone	C_13_H_22_O	23.89	1.34 ± 0.04 ^A,b^	0.98 ± 0.08 ^B,c^	0.33 ± 0.02 ^C,b^	1.97 ± 0.12 ^B,a^	2.19 ± 0.13 ^A,a^	0.18 ± 0.01 ^C,c^	1.85 ± 0.28 ^A,a^	1.51 ± 0.09 ^B,b^	1.19 ± 0.06 ^B,a^
12	593-08-8	2-Tridecanone	C_13_H_26_O	24.95	-	-	-	-	-	0.27 ± 0 ^b^	-	-	0.45 ± 0 ^a^
13	38818-55-2	4,7,9-Megastigmatrien-3-one	C_13_H_18_O	27.00	-	-	-	0.32 ± 0.01 ^A,a^	0.27 ± 0.03 ^B,a^	0.09 ± 0 ^C,b^	0.31 ± 0 ^A,a^	0.29 ± 0.01 ^A,a^	0.28 ± 0.02 ^A,a^
14	119-61-9	Benzophenone	C_13_H_10_O	28.11	0.11 ± 0 ^A,a^	0.04 ± 0 ^C,c^	0.09 ± 0 ^B,a^	0.05 ± 0 ^B,b^	0.07 ± 0.01 ^A,b^	0.03 ± 0 ^C,c^	0.05 ± 0 ^C,b^	0.08 ± 0 ^A,a^	0.07 ± 0 ^B,b^
**Total**		**Ketones**			**12.14 ± 0.31 ^A,a^**	**12.08 ± 0.16 ^A,b^**	**7.62 ± 0.25 ^B,c^**	**10.36 ± 0.14 ^B,b^**	**20.39 ± 0.85 ^A,a^**	**11.22 ± 0.34 ^B,b^**	**8.22 ± 0.09 ^B,c^**	**12.24 ± 0.43 ^A,b^**	**12.30 ± 0.06 ^A,a^**
15	98-01-1	Furfural	C_5_H_4_O_2_	6.24	13.71 ± 0.25	0.17 ± 0.01	-	-	-	-	-	-	-
16	18829-55-5	2-Heptenal, (E)-	C_7_H_12_O	9.78	-	0.31 ± 0.02	-	-	-	-	0.28 ± 0.02 ^B^	-	5.93 ± 0.12 ^A^
17	2548-87-0	2-Octenal, (E)-	C_8_H_14_O	12.90	-	7.85 ± 0.45 ^a^	-	2.14 ± 0.11 ^B,a^	1.04 ± 0.07 ^C,b^	5.22 ± 0.20 ^A,a^	1.80 ± 0.18 ^A,b^	-	1.30 ± 0.07 ^B,b^
18	124-19-6	Nonanal	C_9_H_18_O	14.30	9.36 ± 0.15 ^A^	7.00 ± 0.27 ^B^	1.75 ± 0.17 ^C,c^	-	-	3.73 ± 0.17 ^b^	-	-	12.19 ± 0.47 ^a^
19	60784-31-8	2-Nonenal, (2Z)-	C_9_H_16_O	15.93	0.88 ± 0	-	-	-	-	-	-	-	-
20	18829-56-6	(2E)-2-Nonenal	C_9_H_16_O	15.94	-	0.40 ± 0.02 ^B,b^	0.97 ± 0.07 ^A,a^	-	-	0.75 ± 0.04 ^b^	1.42 ± 0.07 ^A^	1.18 ± 0.20 ^A,B,a^	0.99 ± 0.08 ^B,a^
21	112-31-2	Decyl aldehyde	C_10_H_20_O	17.27	0.72 ± 0.05 ^A,a^	0.24 ± 0.02 ^B,b^	0.61 ± 0.08 ^A,a^	0.22 ± 0.02 ^B,c^	0.23 ± 0.02 ^B,b^	0.32 ± 0.02 ^A,b^	0.42 ± 0.02 ^A,b^	0.30 ± 0.03 ^B,a^	0.25 ± 0.01 ^C,b^
22	5392-40-5	Citral	C_10_H_16_O	18.29	0.37 ± 0.02 ^B,a^	0.42 ± 0.01 ^A,a^	-	0.11 ± 0.01 ^A,c^	0.14 ± 0.01 ^A,c^	-	0.18 ± 0.01 ^B,b^	0.36 ± 0.01 ^A,b^	-
23	123-11-5	p-Anisaldehyde	C_8_H_8_O_2_	18.65	-	-	-	-	2.30 ± 0.10 ^A^	2.75 ± 0.20 ^A^	-	-	-
24	591-31-1	Benzaldehyde, 3-methoxy-	C_8_H_8_O_2_	18.66	-	-	3.17 ± 0.09	-	-	-	-	1.13 ± 0.07 ^B^	1.52 ± 0.14 ^A^
25	3913-81-3	(2E)-2-Decenal	C_10_H_18_O	18.84	-	-	-	-	-	0.27 ± 0 ^b^	-	-	0.45 ± 0 ^a^
26	14371-10-9	Cinnamaldehyde, (E)-	C_9_H_8_O	19.10	-	-	-	0.87 ± 0.03 ^A^	-	0.47 ± 0.02 ^B,a^	-	-	0.44 ± 0.04 ^a^
27	4411-89-6	2-Phenyl-2-butenal	C_10_H_10_O	19.18	1.35 ± 0.06 ^A,a^	0.60 ± 0.04 ^B,c^	1.34 ± 0.11 ^A,a^	0.57 ± 0.03 ^C,b^	0.97 ± 0.02 ^B,a^	1.19 ± 0.10 ^A,a^	0.55 ± 0.03 ^C,b^	0.88 ± 0.01 ^A,b^	0.78 ± 0.06 ^B,b^
28	21391-98-0	1-Cyclohexene-1-carboxaldehyde, 4-(1-methylethyl)-	C_10_H_16_O	19.26	0.61 ± 0 ^A^	0.61 ± 0 ^A^	-	-	-	-	-	-	-
29	26643-91-4	2-Phenyl-4-methyl-2-pentenal	C_12_H_14_O	21.91	-	1.54 ± 0 ^B^	2.92 ± 0.20 ^A^	-	-	-	-	-	-
30	112-54-9	Dodecanal	C_12_H_24_O	22.77	0.38 ± 0	-	-	-	-	-	-	-	-
31	21834-92-4	5-Methyl-2-phenyl-2-hexenal	C_13_H_16_O	24.85	0.74 ± 0.05 ^C,a^	2.11 ± 0.09 ^B,a^	2.76 ± 0.17 ^A,a^	0.45 ± 0.03 ^B,b^	0.89 ± 0.07 ^A,b^	0.79 ± 0.07 ^A,b^	0.26 ± 0.01 ^C,c^	0.63 ± 0.04 ^B,c^	0.81 ± 0.03 ^A,b^
**Total**		**Aldehydes**			**28.12 ± 0.20 ^A,a^**	**21.27 ± 0.09 ^B,a^**	**13.53 ± 0.30 ^C,c^**	**4.36 ± 0.13 ^C,c^**	**5.57 ± 0.28 ^B,b^**	**15.48 ± 0.34 ^A,b^**	**4.90 ± 0.12 ^B,b^**	**4.47 ± 0.30 ^B,c^**	**24.65 ± 0.58 ^A,a^**
32	93-58-3	Methyl benzoate	C_8_H_8_O_2_	14.02	1.01 ± 0.02 ^A^	0.07 ± 0 ^B^	0.04 ± 0 ^C^	-	-	-	-	-	-
33	93-89-0	Ethyl benzoate	C_9_H_10_O_2_	16.27	1.18 ± 0.05 ^A,a^	0.46 ± 0.04 ^C,b^	0.61 ± 0.08 ^B^	1.10 ± 0.03 ^A,b^	0.48 ± 0.01 ^B,b^	-	0.60 ± 0.03 ^B,c^	1.12 ± 0.02 ^A,a^	-
34	101-97-3	Ethyl phenylacetate	C_10_H_12_O_2_	18.39	0.73 ± 0.02 ^B,a^	0.21 ± 0.02 ^C,c^	0.85 ± 0.06 ^A,a^	0.41 ± 0.01 ^B,c^	0.49 ± 0.01 ^B,b^	0.70 ± 0.08 ^A,b^	0.48 ± 0.02 ^B,b^	0.65 ± 0.05 ^A,a^	0.16 ± 0.01 ^C,c^
35	103-45-7	Phenethyl acetate	C_10_H_12_O_2_	18.73	-	-	-	-	-	0.43 ± 0.06 ^a^	0.18 ± 0.01 ^B^	0.26 ± 0.01 ^A^	0.19 ± 0.02 ^B,b^
36	80-26-2	α- Terpinyl acetate	C_12_H_2_0O_2_	21.26	0.32 ± 0.01 ^B,c^	0.31 ± 0.04 ^B,b^	0.61 ± 0.06 ^A^	0.68 ± 0.03 ^A,a^	0.38 ± 0.02 ^B,a^	-	0.55 ± 0.04 ^A,b^	0.39 ± 0.03 ^B,a^	-
37	6846-50-0	2,2,4-Trimethyl-1,3-pentanediol diisobutyrate	C_16_H_30_O_4_	21.33	-	-	-	7.43 ± 0.33 ^A,a^	2.93 ± 0.14 ^B,b^	1.55 ± 0.07 ^C,b^	6.22 ± 0.10 ^A,b^	5.32 ± 0.02 ^B,a^	5.33 ± 0.09 ^B,a^
38	104-61-0	2(3H)-Furanone, dihydro-5-pentyl-	C_9_H_16_O_2_	21.58	3.84 ± 0.15 ^A,b^	1.19 ± 0.11 ^C,c^	3.35 ± 0.03 ^B,a^	4.91 ± 0.04 ^A,a^	4.01 ± 0.04 ^B,a^	0.99 ± 0.05 ^C,b^	3.70 ± 0.10 ^A,b^	2.14 ± 0.18 ^B,b^	3.58 ± 0.25 ^A,a^
39	77-68-9	2-Methyl-propanoic Acid 3-Hydroxy-2,2,4-trimethylpentyl Ester	C_12_H_24_O_3_	21.88	1.76 ± 0.12 ^A,b^	0.75 ± 0.01 ^C,c^	1.32 ± 0.12 ^B,c^	1.41 ± 0.10 ^C,c^	1.67 ± 0.15 ^B,b^	2.16 ± 0.04 ^A,a^	2.29 ± 0.06 ^B,a^	2.64 ± 0.06 ^A,a^	1.59 ± 0.15 ^C,b^
40	131-11-3	Dimethyl phthalate	C_10_H_10_O_4_	23.95	0.47 ± 0.04 ^A,b^	0.24 ± 0.02 ^B,c^	0.10 ± 0.01 ^C,c^	0.87 ± 0.06 ^A,a^	0.42 ± 0.03 ^B,b^	0.20 ± 0.01 ^C,b^	0.47 ± 0.04 ^C,b^	0.65 ± 0.03 ^A,a^	0.58 ± 0.04 ^A,a^
41	17092-92-1	Dihydroactinidiolide	C_11_H_16_O_2_	25.86	0.16 ± 0.01 ^A,a^	0.11 ± 0.01 ^B,b^	0.04 ± 0 ^C,b^	0.08 ± 0 ^B,c^	0.12 ± 0.01 ^A,b^	0.03 ± 0 ^C,b^	0.14 ± 0 ^B,b^	0.19 ± 0.01 ^A,a^	0.15 ± 0.01 ^B,a^
42	84-66-2	Diethyl phthalate	C_12_H_14_O_4_	27.34	0.09 ± 0	-	-	-	-	-	-	-	-
43	18679-18-0	(Z)-Dihydro-5-(2-octenyl)furan-2(3H)-one	C_12_H_20_O_2_	28.72	0.24 ± 0.01 ^B,b^	0.19 ± 0.01 ^B,b^	0.53 ± 0.07 ^A,a^	0.30 ± 0.01 ^A,a^	0.33 ± 0.02 ^A,a^	0.11 ± 0.01 ^B,c^	0.25 ± 0.02 ^C,b^	0.32 ± 0.03 ^B,a^	0.43 ± 0.02 ^A,b^
44	2305-05-7	4-Dodecanolide	C_12_H_22_O_2_	29.23	0.05 ± 0 ^C,c^	0.09 ± 0.01 ^B,c^	0.30 ± 0.02 ^A,a^	0.19 ± 0.01 ^A,b^	0.12 ± 0.01 ^B,b^	0.09 ± 0 ^C,c^	0.30 ± 0.02 ^A,a^	0.25 ± 0.02 ^B,a^	0.20 ± 0.02 ^C,b^
45	124-10-7	Methyl tetradecanoate	C_15_H_30_O_2_	30.19	0.01 ± 0 ^C,c^	0.02 ± 0 ^B,c^	0.10 ± 0.01 ^A,a^	0.04 ± 0 ^B,b^	0.03 ± 0 ^C,b^	0.05 ± 0 ^A,b^	0.05 ± 0 ^B,a^	0.06 ± 0 ^A,a^	0.06 ± 0 ^A,b^
46	124-06-1	Tetradecanoic acid, ethyl ester	C_16_H_32_O_2_	31.89	-	-	-	0.07 ± 0.01 ^A,b^	0.03 ± 0 ^B^	-	0.09 ± 0.01 ^A,a^	0.06 ± 0 ^B^	-
47	112-39-0	Methyl palmitate	C_17_H_34_O_2_	34.54	0.85 ± 0.01 ^B,a^	0.31 ± 0.02 ^C,b^	1.38 ± 0.06 ^A,a^	0.71 ± 0.04 ^B,b^	0.29 ± 0.02 ^C,b^	0.85 ± 0.07 ^A,c^	0.35 ± 0.03 ^C,c^	0.54 ± 0.01 ^B,a^	1.01 ± 0.05 ^A,b^
48	84-74-2	Dibutyl phthalate	C_16_H_22_O_4_	35.13	0.23 ± 0.01 ^A,a^	0.03 ± 0 ^C,a^	0.20 ± 0.01 ^B,a^	0.05 ± 0 ^A,b^	0.02 ± 0 ^C,b^	0.03 ± 0 ^B,c^	0.04 ± 0 ^A,b^	0.03 ± 0 ^B,a^	0.04 ± 0 ^A,b^
49	628-97-7	Palmitic acid ethyl ester	C_18_H_3_6O_2_	35.53	2.01 ± 0.17 ^A^	1.07 ± 0.02 ^B,c^	0.32 ± 0.03 ^C,c^	1.54 ± 0.07 ^A^	1.18 ± 0.03 ^C,b^	1.34 ± 0.09 ^B,b^	1.56 ± 0.11 ^B^	1.53 ± 0.05 ^B,a^	1.97 ± 0.07 ^A,a^
50	112-63-0	Methyl linoleate	C_19_H_34_O_2_	36.74	-	-	1.23 ± 0.02 ^b^	-	-	0.30 ± 0.02 ^c^	-	-	1.43 ± 0.06 ^a^
51	13058-52-1	Methyl (9Z,11E)-9,11-octadecadienoate	C_19_H_34_O_2_	36.75	0.42 ± 0.06 ^ab^	-	-	0.35 ± 0.02 ^b^	-	-	0.46 ± 0.03 ^A,a^	0.31 ± 0.01 ^B^	-
52	544-35-4	Linoleic acid ethyl ester	C_20_H_36_O_2_	37.47	0.88 ± 0.02 ^A,a^	0.37 ± 0.04 ^B,b^	0.28 ± 0.02 ^C,c^	0.75 ± 0.03 ^A,b^	0.35 ± 0.02 ^B,b^	0.83 ± 0.07 ^A,b^	0.52 ± 0.06 ^C,c^	0.74 ± 0.03 ^B,a^	4.13 ± 0.26 ^A,a^
53	6114-18-7	Elaidic acid ethyl ester	C_20_H_38_O_2_	37.52	0.22 ± 0.02 ^B,c^	0.18 ± 0.02 ^B,b^	0.34 ± 0.03 ^A,c^	0.66 ± 0.04 ^A,a^	0.20 ± 0.01 ^B,b^	0.66 ± 0.05 ^A,b^	0.56 ± 0.05 ^B,b^	0.36 ± 0.02 ^C,a^	1.85 ± 0.08 ^A,a^
54	111-62-6	Ethyl oleate	C_20_H_38_O_2_	37.53	-	0.52 ± 0	-	-	-	0.96 ± 0	-	-	-
**Total**		**Esters**			**14.48 ± 0.23 ^A,c^**	**6.12 ± 0.10 ^C,c^**	**11.59 ± 0.23 ^B,b^**	**21.54 ± 0.63 ^A,a^**	**13.03 ± 0.30 ^B,b^**	**11.28 ± 0.12 ^C,b^**	**18.78 ± 0.26 ^B,b^**	**17.56 ± 0.22 ^C,a^**	**22.70 ± 0.43 ^A,a^**
55	90-05-1	2-Methoxyphenol	C_7_H_8_O_2_	13.84	3.88 ± 0.14 ^A,a^	0.31 ± 0.01 ^B^	4.04 ± 0.12 ^A,a^	1.33 ± 0.08 ^b^	-	-	-	-	0.83 ± 0.50 ^b^
56	2628-17-3	4-Vinylphenol	C_8_H_8_O	17.58	-	-	-	7.53 ± 0.30	-	-	-	-	-
57	621-58-9	Phenol, 5-ethenyl-2-methoxy-	C_9_H_10_O_2_	20.30	-	-	-	-	-	2.62 ± 0.1	-	-	-
58	97-53-0	Eugenol	C_10_H_12_O_2_	21.46	0.57 ± 0.03 ^A,c^	0.51 ± 0.05 ^A,c^	0.20 ± 0.02 ^B,c^	0.80 ± 0.04 ^A,b^	0.59 ± 0.02 ^B,b^	0.76 ± 0.07 ^A,b^	0.92 ± 0.08 ^A,a^	0.69 ± 0.02 ^B,a^	0.92 ± 0.07 ^A,a^
**Total**		**Phenols**			**4.45 ± 0.17 ^A,b^**	**0.82 ± 0.05 ^B,a^**	**4.24 ± 0.11 ^A,a^**	**9.66 ± 0.36 ^A,a^**	**0.59 ± 0.02 ^C,c^**	**3.38 ± 0.17 ^B,b^**	**0.92 ± 0.08 ^B,c^**	**0.69 ± 0.02 ^B,b^**	**1.75 ± 0.47 ^A,c^**
59	100-51-6	Benzyl alcohol	C_7_H_8_O	12.19	-	2.12 ± 0.16	-	-	-	0.10 ± 0.02	0.44 ± 0.05	-	-
60	18409-17-1	2-Octen-1-ol, (E)-	C_8_H_16_O	13.19	-	-	-	0.29 ± 0	-	-	-	0.33 ± 0	-
61	78-70-6	Linalool	C_10_H_18_O	14.16	5.34 ± 0.19 ^A,a^	0.79 ± 0.04 ^C,b^	2.96 ± 0.28 ^B,b^	2.28 ± 0.14 ^A,b^	1.68 ± 0.15 ^B,a^	2.46 ± 0.05 ^A,c^	1.56 ± 0.06 ^B,c^	1.55 ± 0.03 ^B,a^	6.55 ± 0.09 ^A,a^
62	60-12-8	Phenylethyl Alcohol	C_8_H_10_O	14.55	2.00 ± 0.12 ^C^	3.97 ± 0.07 ^A^	2.36 ± 0.19 ^B,b^	1.44 ± 0.12 ^B^	-	2.83 ± 0.16 ^A,a^	-	-	-
63	10340-23-5	(Z)-3-Nonen-1-ol	C_9_H_18_O	15.75	3.57 ± 0.16 ^B,c^	1.68 ± 0.14 ^C,b^	4.34 ± 0.44 ^A,a^	5.84 ± 0.38 ^B,a^	7.04 ± 0.27 ^A,a^	2.94 ± 0.24 ^C,b^	4.84 ± 0.07 ^B,b^	6.71 ± 0.30 ^A,a^	2.16 ± 0.02 ^C,c^
64	20126-76-5	(-)-Terpinen-4-ol	C_10_H_18_O	16.46	6.09 ± 0.47 ^A,b^	2.68 ± 0.08 ^B,b^	5.47 ± 0.55 ^A,b^	6.49 ± 0.51 ^B,b^	8.66 ± 0.71 ^A,a^	8.08 ± 0.38 ^A,a^	17.04 ± 1.18 ^A,a^	9.41 ± 0.33 ^B,a^	2.46 ± 0.16 ^C,c^
65	10482-56-1	(S)-(-)-α-Terpineol	C_10_H_18_O	16.85	5.30 ± 0.13 ^A,b^	1.62 ± 0.09 ^B,c^	-	1.20 ± 0.04 ^C,c^	2.34 ± 0.07 ^B,b^	5.47 ± 0.18 ^A,a^	6.04 ± 0.17 ^A,a^	4.66 ± 0.19 ^B,a^	1.85 ± 0.12 ^C,b^
66	98-55-5	α-Terpineol	C_10_H_18_O	16.87	-	8.40 ± 0.85 ^A^	3.89 ± 0.13 ^B^	-	-	-	1.67 ± 0.09	-	-
67	17301-29-0	Undecane,3,7-dimethyl-	C_13_H_28_	19.36	-	-	-	0.37 ± 0 ^B^	-	0.94 ± 0.0 ^A^	-	-	-
**Total**		**Alcohols**			**22.31 ± 0.42 ^A,b^**	**21.25 ± 0.78 ^A,b^**	**19.02 ± 1.27 ^B,b^**	**17.91 ± 0.39 ^C,c^**	**19.72 ± 0.29 ^B,c^**	**22.82 ± 0.20 ^A,a^**	**31.58 ± 1.49 ^A,a^**	**22.68 ± 0.25 ^B,a^**	**13.03 ± 0.18 ^C,c^**
68	65-85-0	Benzoic acid	C_7_H_6_O_2_	16.00	2.70 ± 0.22 ^B^	0.47 ± 0.04 ^C^	9.30 ± 0.36 ^A^	-	-	-	-	-	-
69	124-07-2	Octanoic acid	C_8_H_16_O_2_	16.30	-	0.44 ± 0.03 ^B,b^	1.86 ± 0.07 ^A,b^	0.74 ± 0 ^C^	3.49 ± 0.30 ^A,a^	2.55 ± 0.22 ^B,a^	-	-	-
70	112-05-0	Nonanoic acid	C_9_H_18_O_2_	18.98	1.71 ± 0.08 ^C,b^	1.98 ± 0.16 ^B,a^	3.34 ± 0.13 ^A,a^	1.39 ± 0.11 ^B,c^	1.74 ± 0.18 ^A,a^	2.03 ± 0.15 ^A,b^	2.32 ± 0.16 ^A,a^	1.31 ± 0.02 ^B,b^	0.99 ± 0.05 ^C,c^
71	31642-67-8	8-Nonenoic Acid	C_9_H_16_O_2_	19.21	0.33 ± 0	-	-	-	-	-	-	-	-
72	501-52-0	Hydrocinnamic acid	C_9_H_10_O_2_	20.78	-	-	1.47 ± 0	-	-	-	-	-	-
73	544-63-8	Myristic acid	C_14_H_28_O_2_	30.95	0.14 ± 0 ^B^	-	2.29 ± 0.14 ^A,a^	-	-	0.02 ± 0 ^b^	-	-	-
74	57-10-3	Palmitic acid	C_16_H_32_O_2_	35.03	1.53 ± 0.05 ^A,a^	0.18 ± 0.01 ^C,c^	0.94 ± 0.06 ^B,c^	0.67 ± 0.03 ^B,b^	0.43 ± 0.03 ^C,a^	2.28 ± 0.08 ^A,a^	0.51 ± 0.05 ^B,c^	0.37 ± 0.02 ^B,b^	2.09 ± 0.11 ^A,b^
75	544-71-8	(9E,11E)-9,11-Octadecadienoic acid	C_18_H_32_O_2_	36.22	-	-	-	0.50 ± 0.02 ^A,a^	0.30 ± 0.03 ^B,b^	0.08 ± 0 ^C,b^	0.35 ± 0.03 ^B,b^	0.36 ± 0.02 ^B,a^	0.95 ± 0.04 ^A,a^
76	2420-56-6	10E,12Z-Octadecadienoic acid	C_18_H_32_O_2_	37.14	-	-	-	-	-	0.52 ± 0.0	-	-	-
77	57-11-4	Octadecanoic acid	C_18_H_36_O_2_	37.42	-	-	0.31 ± 0.0	-	-	-	-	-	-
**Total**		**Acids**			**6.41 ± 0.20 ^B,a^**	**3.07 ± 0.17 ^C,b^**	**19.52 ± 0.30 ^A,a^**	**3.31 ± 0.12 ^C,b^**	**5.95 ± 0.48 ^B,a^**	**7.47 ± 0.10 ^A,b^**	**3.19 ± 0.15 ^B,b^**	**2.04 ± 0.05 ^C,c^**	**4.03 ± 0.16 ^A,c^**
78	100-41-4	Ethylbenzene	C_8_H1_0_	7.23	1.40 ± 0.09 ^b^	-	-	-	-	-	2.38 ± 0.15 ^A,a^	0.13 ± 0.01 ^B^	-
79	99-87-6	p-cymene	C_10_H_14_	11.90	2.39 ± 0.19 ^b^	-	-	3.16 ± 0.09 ^A,a^	-	1.24 ± 0.03 ^B,b^	0.30 ± 0.04 ^B,c^	0.29 ± 0.02 ^B^	2.02 ± 0.04 ^A,a^
80	91-20-3	Naphthalene	C_10_H_8_	16.62	0.71 ± 0.03 ^A,a^	0.59 ± 0.05 ^B,a^	0.67 ± 0.06 ^A,B,b^	0.66 ± 0.05 ^A,a^	0.52 ± 0.07 ^B,a^	0.33 ± 0.03 ^C,c^	0.59 ± 0.02 ^B,b^	0.63 ± 0.06 ^B,a^	0.95 ± 0.04 ^A,a^
81	31295-56-4	2,6,11-Trimethyldodecane	C_15_H_32_	20.86	-	-	-	-	-	0.05 ± 0 ^b^	0.87 ± 0.06 ^A^	0.57 ± 0.04 ^B^	0.50 ± 0.03 ^B,a^
82	99-86-5	α-Terpinene	C_10_H_1_6	21.26	-	-	-	-	0.29 ± 0.01 ^B^	0.59 ± 0.05 ^A,a^	-	-	0.41 ± 0.02 ^b^
83	644-30-4	alpha-Curcumene	C_15_H_2_2	24.68	-	-	-	-	-	0.08 ± 0 ^b^	0.19 ± 0.01 ^A^	0.18 ± 0 ^A^	0.13 ± 0.01 ^B,a^
**Total**		**Hydrocarbons**			**4.50 ± 0.28 ^A,a^**	**0.59 ± 0.05 ^B,c^**	**0.67 ± 0.06 ^B,c^**	**3.82 ± 0.12 ^A,b^**	**0.80 ± 0.07 ^C,b^**	**2.29 ± 0.04 ^B,b^**	**4.33 ± 0.20 ^A,a^**	**1.80 ± 0.11 ^C,a^**	**4.00 ± 0.06 ^B,a^**
84	3268-49-3	Methional	C_4_H_8_OS	8.29	0.14 ± 0 ^B^	19.08 ± 0.3 ^A^	-	-	-	-	-	-	-
85	108-50-9	Pyrazine, 2,6-dimethyl-	C_6_H_8_N_2_	8.47	-	-	-	-	-	3.86 ± 0.2	-	-	-
86	104-93-8	4-Methylanisole	C_8_H_10_O	9.13	-	-	0.64 ± 0	-	-	-	-	-	-
87	3777-69-3	2-Pentylfuran	C_9_H_14_O	10.88	-	0.43 ± 0.0	-	-	-	6.55 ± 0.4	-	-	-
88	13925-09-2	2-Methyl-6-vinylpyrazine	C_7_H_8_N_2_	11.56	-	-	-	0.67 ± 0.04	0.43 ± 0.03 ^b^	-	0.21 ± 0.01 ^C^	0.89 ± 0.07 ^A,a^	0.61 ± 0.04 ^B^
89	13925-05-8	Pyrazine, 2-methyl-5-(1-methylethyl)-	C_8_H_12_N_2_	12.56	-	-	0.79 ± 0.0	-	-	-	-	-	-
90	1072-83-9	2-Acetylpyrrole	C_6_H_7_NO	12.92	1.36 ± 0.04 ^C,a^	2.51 ± 0.05 ^A,a^	1.73 ± 0.13 ^B,b^	0.17 ± 0.01 ^C,c^	0.80 ± 0.05 ^B,b^	2.75 ± 0.17 ^A,a^	0.31 ± 0.02 ^C,b^	0.40 ± 0.04 ^B,c^	0.53 ± 0.05 ^A,c^
91	13067-27-1	Pyrazine, 2,6-diethyl-	C_8_H_12_N_2_	13.54	-	-	0.65 ± 0.04 ^a^	1.35 ± 0.11	-	-	-	-	0.49 ± 0.03 ^b^
92	1124-11-4	Tetramethylpyrazine	C_8_H_12_N_2_	13.75	-	-	-	0.45 ± 0.04 ^B,b^	4.48 ± 0.34 ^A^	-	1.17 ± 0.02 ^a^	-	-
93	637-69-4	4-Methoxystyrene	C_9_H_10_O	15.23	0.88 ± 0.05 ^A,b^	0.83 ± 0.08 ^A,a^	0.47 ± 0.05 ^B,b^	1.11 ± 0.03 ^B,a^	0.63 ± 0.06 ^C,b^	1.34 ± 0.12 ^A,a^	0.89 ± 0.03 ^B,b^	0.88 ± 0.1 ^B,a^	1.27 ± 0.05 ^A,a^
94	140-67-0	Estragole	C_10_H_12_O	17.08	5.11 ± 0.21 ^C,b^	11.74 ± 0.90 ^B,c^	17.96 ± 1.21 ^A,a^	22.78 ± 1.24 ^B,a^	24.92 ± 0.84 ^A,b^	9.57 ± 0.23 ^C,c^	23.06 ± 0.86 ^B,a^	33.69 ± 0.90 ^A,a^	12.74 ± 1.26 ^C,b^
95	496-16-2	2,3-Dihydrobenzofuran	C_8_H_8_O	17.59	-	-	-	-	-	0.69 ± 0	-	-	-
96	13679-41-9	3-Phenylfuran	C_10_H_8_O	17.73	-	-	1.22 ± 0.03 ^b^	1.74 ± 0.11 ^B,a^	2.28 ± 0.11 ^A,a^	-	1.07 ± 0.09 ^C,b^	1.52 ± 0.01 ^B,b^	1.66 ± 0.06 ^A,a^
97	95-16-9	Benzothiazole	C_7_H_5_NS	17.79	0.09 ± 0 ^C,c^	0.21 ± 0.02 ^B,c^	0.35 ± 0.01 ^A,b^	0.78 ± 0.01 ^B,b^	0.41 ± 0.05 ^C,b^	1.29 ± 0.02 ^A,a^	1.35 ± 0.06 ^A,a^	1.13 ± 0.02 ^B,a^	0.23 ± 0.01 ^C,c^
**Total**		**Others**			**7.58 ± 0.20 ^C,b^**	**34.80 ± 0.43 ^A,b^**	**23.81 ± 0.99 ^B,b^**	**29.04 ± 1.16 ^B,a^**	**33.96 ± 0.68 ^A,b^**	**26.05 ± 0.12 ^C,a^**	**28.07 ± 0.77 ^B,a^**	**38.52 ± 0.73 ^A,a^**	**17.55 ± 1.27 ^C,c^**

Note: The means (n = 3) for the same salt content with different uppercase letters (A–C) and for the same duration of fermentation with different lowercase letters (a–c) differed significantly (*p* < 0.05).

**Table 3:** Alpha diversity indices of microbial communities of watermelon soybean paste from different salt contents.

	Diversity Index				Abundance Index		Observed Species	
	Shannon		Simpson		Chao1			
	Bacteria	Fungi	Bacteria	Fungi	Bacteria	Fungi	Bacteria	Fungi
**X30**	4.75 ± 0.05 ^A,c^	2.19 ± 0.08 ^C,a^	0.93 ± 0 ^A,b^	0.48 ± 0.02 ^C,a^	245.65 ± 19.49 ^A,b^	119.49 ± 8.48 ^B,a^	231.17 ± 14.69 ^A,c^	116.83 ± 7.68 ^B,a^
**X45**	4.28 ± 0.03 ^C,c^	2.71 ± 0.14 ^B,a^	0.89 ± 0 ^C,b^	0.66 ± 0.02 ^B,a^	181.20 ± 3.66 ^B,c^	111.61 ± 7.6 ^C,a^	168.83 ± 6.05 ^B,c^	110.00 ± 7.27 ^B,a^
**X60**	4.48 ± 0.03 ^B,c^	4.17 ± 0.04 ^A,a^	0.91 ± 0 ^B,c^	0.88 ± 0 ^A,a^	174.44 ± 13.2 ^B,c^	147.31 ± 6.33 ^A,a^	164.83 ± 11.41 ^B,c^	146.83 ± 6.11 ^A,a^
**Y30**	5.29 ± 0.01 ^C,b^	1.14 ± 0.08 ^AB,b^	0.95 ± 0 ^A,a^	0.24 ± 0.02 ^A,b^	265.70 ± 7.11 ^C,b^	75.21 ± 5.53 ^A,b^	249.33 ± 6.38 ^C,b^	74.50 ± 5.36 ^B,b^
**Y45**	5.47 ± 0.09 ^A,a^	1.03 ± 0.10 ^B,b^	0.95 ± 0 ^A,a^	0.22 ± 0.02 ^A,b^	377.98 ± 39.84 ^A,a^	59.17 ± 5.23 ^B,b^	353.33 ± 41.15 ^A,a^	59.17 ± 5.23 ^C,b^
**Y60**	5.35 ± 0.03 ^B,a^	1.17 ± 0.12 ^A,b^	0.95 ± 0 ^A,a^	0.25 ± 0.03 ^A,b^	333.75 ± 10.33 ^B,a^	81.19 ± 3.90 ^A,b^	313.00 ± 10.83 ^B,a^	81.17 ± 3.87 ^A,b^
**Z30**	5.38 ± 0.02 ^A,a^	0.99 ± 0.10 ^A,c^	0.95 ± 0 ^A,a^	0.21 ± 0.02 ^A,c^	319.98 ± 19.62 ^A,a^	67.67 ± 5.65 ^A,b^	297.17 ± 17.35 ^A,a^	67.67 ± 5.65 ^A,b^
**Z45**	5.25 ± 0.02 ^B,b^	0.68 ± 0.12 ^B,c^	0.95 ± 0 ^A,a^	0.14 ± 0.03 ^B,c^	292.13 ± 15.27 ^B,b^	53.55 ± 7.33 ^B,b^	275.17 ± 11.44 ^B,b^	53.50 ± 7.29 ^B,b^
**Z60**	5.06 ± 0.05 ^C,b^	0.70 ± 0.09 ^B,c^	0.94 ± 0 ^B,b^	0.14 ± 0.02 ^B,c^	213.39 ± 8.66 ^C,b^	69.13 ± 7.17 ^A,c^	204.17 ± 8.42 ^C,b^	67.67 ± 6.62 ^A,c^

Note: The means (n = 6) for the same salt content with different uppercase letters (A–C) and for the same duration of fermentation with different lowercase letters (a–c) differed significantly (*p* < 0.05).

## Data Availability

The original contributions presented in this study are included in the article/Supplementary Material. Further inquiries can be directed to the corresponding authors.

## Supplementary Materials

The following supporting information can be downloaded at: https://www.mdpi.com/article/10.3390/foods15152767/s1. Figure S1: 200-permutation testing analysis of volatile aroma compounds of watermelon soybean paste from different salt contents. (A: 200-permutation testing of Group X of watermelon soybean paste in three fermentation stages (30 d, 45 d, 60 d), B: 200-permutation testing of Group Y of watermelon soybean paste in three fermentation stages (30 d, 45 d, 60 d), C: 200-permutation testing of Group Z of watermelon soybean paste in three fermentation stages (30 d, 45 d, 60 d), D: 200-permutation testing of 30 d of watermelon soybean paste from different salt contents, E: 200-permutation testing of 45 d of watermelon soybean paste from different salt contents, F: 200-permutation test-ing of 60 d of watermelon soybean paste from different salt contents).
