# Supplementary material for: Salt Concentration Is Associated with Flavor Divergence and Microbial Succession in Naturally Fermented Watermelon Soybean Paste: Integrating Volatile Metabolite Profiling with Community Dynamics
Source: Foods. 2026 Aug 6;15(15):2767. doi: 10.3390/foods15152767 (PMC13464426; doi:10.3390/foods15152767)
Supplement: Supplementary file 1 [file foods-15-02767-s001.zip › foods-4408860-supplementary.pdf]

Supplemental Information

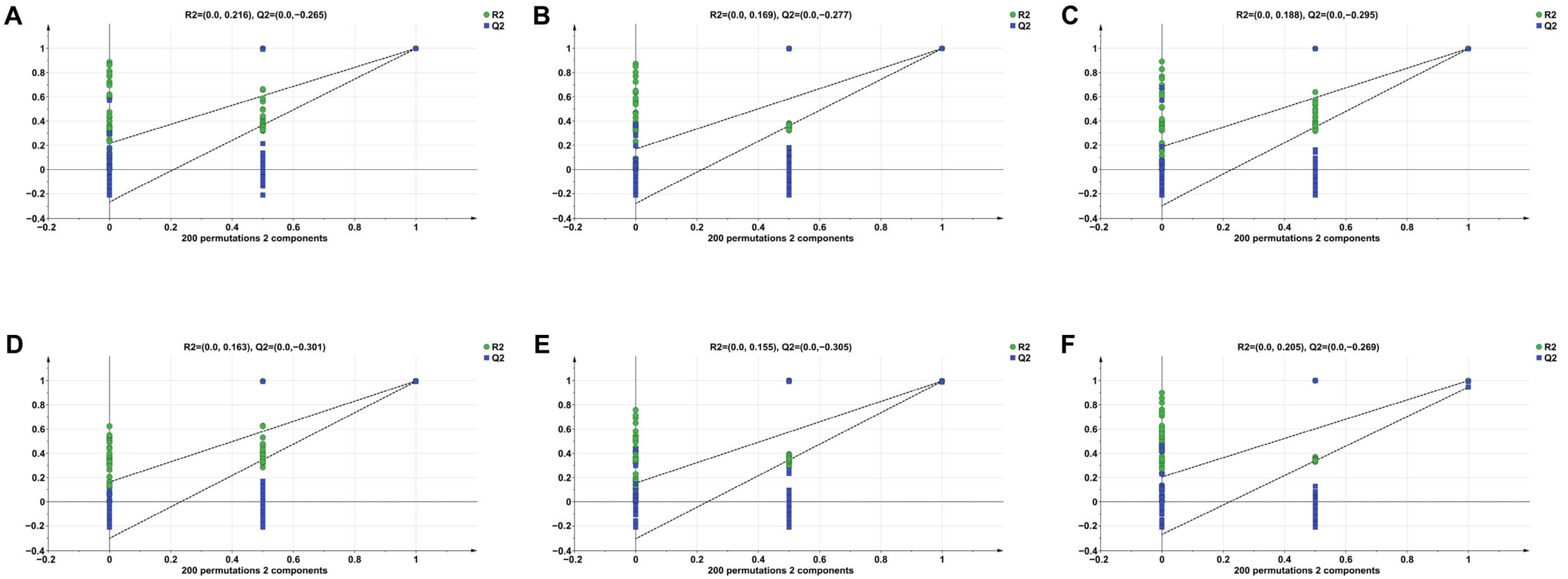

Figure S1. 200-permutation testing analysis of volatile aroma compounds of watermelon soybean paste from different salt contents. (A: 200-permutation testing of Group X of watermelon soybean paste in three fermentation stages (30 d, 45 d, 60 d), B: 200-permutation testing of Group Y of watermelon soybean paste in three fermentation stages (30 d, 45 d, 60 d), C: 200-permutation testing of Group Z of watermelon soybean paste in three fermentation stages (30 d, 45 d, 60 d), D: 200-permutation testing of 30 d of watermelon soybean paste from different salt contents, E: 200-permutation testing of 45 d of watermelon soybean paste from different salt contents, F: 200-permutation testing of 60 d of watermelon soybean paste from different salt contents).
